# The Efficacy of High-Dose Pulse Therapy vs. Low-Dose Intravenous Methylprednisolone on Severe to Critical COVID-19 Clinical Outcomes: A Randomized Clinical Trial

**DOI:** 10.5812/ijpr-137838

**Published:** 2023-08-26

**Authors:** Zahra Sahraei, Parnaz Panahi, Kouroush Solhjoukhah, Maryam Mesbah, Siamak Afaghi, Mahdi Amirdosara, Jamshid Salamzadeh, Farzad Esmaeili Tarki, Ilad Alavi Darazam

**Affiliations:** 1 Department of Clinical Pharmacy, School of Pharmacy, Shahid Beheshti University of Medical Sciences, Tehran, Iran; 2 Prevention of Metabolic Disorders Research Center, Research Institute for Endocrine Sciences, Shahid Beheshti University of Medical Sciences, Tehran,Iran; 3 Anesthesiology Research Center, Loghman Hakim Hospital, Shahid Beheshti University of Medical Sciences, Tehran, Iran; 4 School of Pharmacy, Shahid Beheshti University of Medical Sciences, Tehran, Iran; 5 Research Institute of Internal Medicine, Shahid Modarres Hospital, Shahid Behehsti University of Medical Sciences,Tehran, Iran; 6 Infectious Diseases and Tropical Medicine Research Center, Shahid Beheshti University of Medical Sciences, Tehran, Iran

**Keywords:** Methylprednisolone, Steroid, COVID-19, SARS-CoV-2, Pulse Therapy

## Abstract

**Background:**

It remains unclear which formulation of the corticosteroid regimen has the optimum efficacies on COVID-19 pneumonia.

**Objectives:**

The aim of this study was to compare the clinical outcomes of 2 different regimens in the treatment of acute respiratory distress syndrome (ARDS) caused by COVID-19: Methylprednisolone at a dose of 1 mg/kg every 12 hours (low-dose group) and 1000 mg/day pulse therapy for 3 days following 1 mg/kg methylprednisolone every 12 hours (high-dose group).

**Methods:**

In this randomized clinical trial, patients with mild to moderate ARDS due to COVID-19 were randomly assigned to receive either low-dose (n = 47) or high-dose (n = 48) intravenous methylprednisolone regimens. Two groups were matched for age, gender, body mass index (BMI), comorbidities, leukocytes, lymphocytes, neutrophil/lymphocyte, platelet, hemoglobin, and inflammatory markers (erythrocyte sedimentation rate [ESR], C-reactive protein [CRP], and ferritin). Both regimens were initiated upon admission and continued for 10 days. The clinical outcome and secondary complications were evaluated.

**Results:**

Evaluating in-hospital outcomes, no difference was revealed in the duration of intensive care unit (ICU) stays (5.4 ± 4.6 vs. 4.5 ± 4.9; P = 0.35), total hospital stays (8 ± 3.1 vs. 6.9 ± 3.4; P = 0.1), requirement rate for invasive ventilation (29.2% vs. 36.2%; P = 0.4) or non-invasive ventilation (16.6% vs 23.4%; P = 0.4), and hemoperfusion (16.6% vs 11.3%; P = 0.3) between the low- and high-dose groups. There was no significant difference in fatality due to ARDS (29.2% vs. 38.3%; P = 0.3) and septic shock (4.2% vs. 6.4%; P = 0.3) between the low- and high-dose groups. Patients in the high-dose group had significantly higher bacterial pneumonia co-infection events compared with those in the low-dose group (18.7% vs 10.6%; P = 0.01).

**Conclusions:**

The use of adjuvant pulse therapy with intravenous methylprednisolone did not result in improved in-hospital clinical outcomes among patients with mild to moderate ARDS due to COVID-19. A higher risk of bacterial pneumonia should be considered in such cases as receiving a higher dose of steroids.

## 1. Background

COVID-19 infection, as a global health concern, has led to a severe crisis and a massive number of deaths since the pandemic emergence (1, 2). Whilst global vaccination has controlled the soaring rate of afflictions and fatalities in the majority of countries, there is still evidence of new waves of disease transmission in some nations. Infection with the virus could result in clinical manifestations with a spectrum of severity from mild to severe and, in some cases, may even result in sudden death (3, 4). The elderly with cardio-metabolic comorbid factors are more likely to experience the worse form of infection with poor clinical outcomes (5, 6). The pathological hyperimmune response following the cytokine storm syndrome due to SARS-CoV-2 infection could play a decisive part in the patient's clinical outcome (7). Such individuals could have endothelial lung injury and micro- and macrovascular thromboembolism events, which cause severe lung damage and multiple organ failures (8). Evidence has shown that corticosteroids may possess anti-inflammatory effects that mitigate hyperimmune response in severe COVID-19 cases, hence preventing the risk of respiratory failure (9-11). The World Health Organization (WHO) firmly advises treating severe COVID-19 infections that require long-term respiratory support with low-dose systemic steroid therapy for a maximum of 10 days (12). Despite various dosages and formulations of steroid therapy being tested on COVID-19 patients during the past 2 and a half years of the pandemic, there is still limited information available about the effectiveness of high-dose corticosteroids as pulse methylprednisolone prior to initial regular steroid therapy (9, 11, 13).

## 2. Objectives

We aimed to evaluate and compare the influence of daily 1 mg/kg methylprednisolone vs. 1000 mg pulse therapy of methylprednisolone before the daily 1 mg/kg methylprednisolone on clinical outcomes, including hospital stay duration, the need for intensive care, and in-hospital mortality among patients diagnosed with mild to moderate acute respiratory distress syndrome (ARDS) caused by COVID-19 pneumonia.

## 3. Methods

### 3.1. Study Design and Participants

This randomized, controlled, parallel-group clinical trial aimed to investigate the effectiveness of a daily regimen of methylprednisolone at 1 mg/kg/12 hours with a 3-day pulse regimen of methylprednisolone at 1000 mg prior to the daily regimen of methylprednisolone at 1 mg/kg/12 hours. The trial was conducted on hospitalized COVID-19 patients with mild to moderate ARDS at a major referral center for COVID-19 patients (Loghman Hakim Hospital) in Tehran, Iran, during the pandemic. The study was initiated on April 2021. Mild to moderate ARDS was defined based on the Berlin criteria. All enrolled patients were older than 18 years, admitted within the first 24 hours of hospitalization when entered into the study, and did not require invasive mechanical ventilation based on their clinical status. The initial suspicion of SARS-CoV-2 pneumonia was conducted based on clinical presentations and chest computed tomography (CT), which was confirmed by a real-time polymerase chain reaction (PCR) test for all individuals. Exclusion criteria were pregnancy or active lactation, identified contra-indication to corticosteroid usage, or a history of dexamethasone allergy, daily intake of oral or intravenous corticosteroid in the past 15 days, expected death within the next 72 hours, the need for invasive ventilation upon admission, uncontrolled hypertension (systolic blood pressure more than 150 mm Hg at the time of admission), being on chronic hemodialysis, having cardiac failure with Ejection Fraction of < 40% or pulmonary edema, severe vasogenic shock (need for norepinephrine infusion > 300 ng/kg/min), acute or chronic kidney failure, hepatic disease, having a terminal disease and life expectancy of under 2 months, not starting prednisolone after 24 hours of admission, and refusal to consent to participate in the trial.

### 3.2. Randomization

The sample size was estimated based on the study by Edalatifard et al. (13). Mortality was considered the primary factor that was assumed to decrease from 40% to 10%. Hence, a total of 32 patients should be enrolled in the study. More cases were considered due to dropout. Participants were randomly assigned in a 1:1 ratio to receive either standard care methylprednisolone at 1 mg/kg/day for 10 days (low-dose group) or a pulse of methylprednisolone at 1000 mg/day for 3 days, followed by 1 mg/kg/day for an additional 10 days (high-dose group). The randomization sequence was generated by computer software in blocks of 6 without stratification. The allocated therapy was not concealed from doctors, patients, or those who evaluated the results (non-blinded study). Further, the National Committee of the Iranian Ministry of Health essentially designed all clinical intervention procedures, including those involving the use of antiviral medicines, antibiotics, additional immunomodulators, anticoagulants, and laboratory tests. The pneumology and infectious diseases departments of the hospital bestowed the choice upon the medical team to decide the initial dosage or eliminate therapeutic options due to the drug's side effects in exceptional cases.

### 3.3. Procedures

A total of 95 participants were enrolled in the study using the block randomization method, and they were divided into 2 groups. One group consisted of 47 individuals who received 1 mg/kg methylprednisolone every day for 10 days, while the other group included 48 patients who received a daily dose of 1000 mg methylprednisolone for 3 days, followed by the same 1 mg/kg daily dose for the next 10 days. Upon hospital admission, the following data were collected for all patients: Demographic information, previous home treatments, coexisting disorders, the time between hospital admission and randomization, the time between initial symptoms and randomization, and laboratory test results at the time of randomization (lymphocytes, leukocytes, C-reactive protein [CRP], D-dimer, lactate dehydrogenase, procalcitonin, serum ferritin, biochemical parameters, and erythrocyte sedimentation rate [ESR]). All patients were monitored all the time by measuring their systolic blood pressure, oxygenic saturation, pulse rate, and electrocardiogram (ECG). The main aim of this study was to evaluate and compare the length of hospital stay, rate of invasive ventilation requirement, hemoperfusion, immunological intervention, and mortality between the 2 groups.

### 3.4. Statistical Analysis

Categorical and continuous variables were presented as case number (percentage) and mean ± SD, respectively. All data were analyzed to be confirmed as being normally distributed by the Shapiro-Wilk test. To compare the categorical variables, the Fisher exact or chi-square test was used when appropriate. The independent *t*-test or Mann-Whitney U test was used to compare the means of continuous variables between the 2 groups when appropriate. Two-sided P values of less than 0.05 were considered statistically significant. All statistical analyses were performed using SPSS version 27 (SPSS Inc, Chicago, IL, USA).

### 3.5. Ethical Considerations

The study was approved by the Ethics Committee of Shahid Beheshti University of Medical Sciences (code: IR.SBMU.PHARMACY.REC.1400.081); in addition, it was registered on the Iranian Registry of Clinical Trials website (code: IRCT20130917014693N13). The study was carried out in conformity with good clinical practice (GCP) principles, the Declaration of Helsinki, regional regulations, and the national protocol for the care of hospitalized COVID-19 patients. All patients provided informed consent before randomization. The authors originally established the trial design, gathered the data, and conducted the analytical evaluations. The manuscript's correctness and data integrity were attested to by all authors, who also read and approved it.

## 4. Results

**Figure 1.**
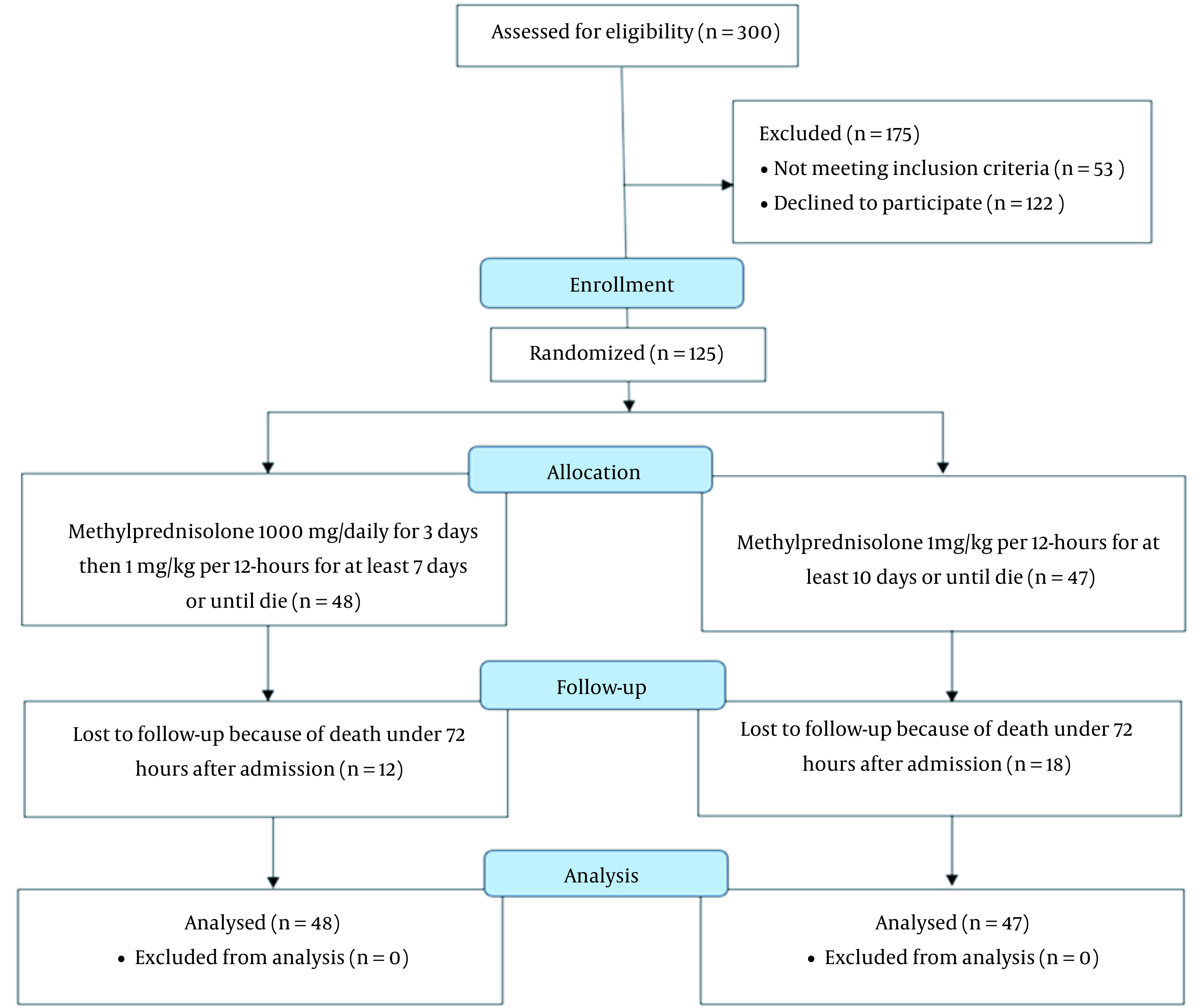
The CONSORT diagram of the study.

The patients were divided into 2 groups: The low-dose methylprednisolone group (which consisted of 48 patients that received a dose of 1 mg/kg every 12 hours) and the high-dose methylprednisolone group, which consisted of 47 patients that received a pulse of 1000 mg pulse methylprednisolone per day for 3 days, followed by a dose of 1 mg/kg every 12 hours (Figure 1). The 2 high- and low-dose groups were matched based on age (54.8 ± 15.0 vs. 60.1 ± 16.4; respectively; P = 0.1), male gender (58.3% vs. 59.6%; P = 0.9), and body mass index (BMI; 28.3 ± 3.6 vs. 28.0 ± 4.2; P = 0.2; Table 1). Further, the high- and low-dose groups were statistically similar in the prevalence of cardiometabolic comorbidities, including diabetes mellitus (16.6% vs. 19.1%; respectively; P = 0.7), hypertension (16.6% vs. 17%; respectively; P = 0.7), dyslipidemia (22.9% vs. 27.6%; respectively; P = 0.5), hypothyroidism (10.4% vs 4.2%; respectively; P = 0.2), and coronary artery disease (14.6% vs 12.8%; respectively; P = 0.7) (Table 1). 

**Table 1.** Baseline Characteristics of the Patients with COVID-19 Pneumonia ^a^

Characteristics	High-Dose Methylprednisolone (N = 48)	Low-Dose Methylprednisolone (N = 47)	P Value
**Sociodemographic**			
Age (y)	54.83 ± 15.05	60.17 ± 16.49	0.103
Gender (male)	29 (58.3)	28 (59.6)	0.933
Body mass index (kg/m^2^)	28.3 ± 3.6	28.0 ± 4.2	0.239
**Comorbidity**			
Diabetes mellitus	8 (16.6)	9 (19.1)	0.756
Hypertension	8 (16.6)	8 (17.0)	0.751
Dyslipidemia	11 (22.9)	13 (27.6)	0.599
Coronary artery disease	7 (14.6)	6 (12.8)	0.799
Hypothyroidism	5 (10.4)	2 (4.2)	0.250
**Laboratorial**			
Leukocytes (cells/mL)	8.4 ± 2.2	8.6 ± 2.5	0.365
Lymphocytes (cells/mL)	1.6 ± 0.76	1.6 ± 0.5	0.325
Neutrophil/lymphocyte ratio	3.62 ± 1.0	3.64 ± 1.2	0.196
Hemoglobin (g/dL)	13.9 ± 1.5	13.7 ± 1.2	0.124
Platelet (cells/mL)	169.2 ± 5.7	166.5 ± 6.2	0.369
Erythrocyte sedimentation rate (mm/h)	32.4 ± 5.3	33.1 ± 4.6	0.485
C-reactive protein (mg/L)	16.3 ± 5.3	14.9 ± 3.6	0.241
Ferritin (ng/mL)	365.2 ± 72.1	375.3 ± 99.2	0.399

^a^ Values are expressed as Mean ± SD or No. (%).

Upon hospital admission, there were no significant differences between the high- and low-dose groups in terms of blood cell count indices, including leukocytes (8.4 vs. 8.6; respectively; P = 0.365), lymphocytes (1.6 vs. 1.6; respectively; P = 0.325), neutrophil to lymphocyte ratio (3.62 vs. 3.64; respectively; P = 0.196), hemoglobin (13.9 vs 13.7; respectively; P = 0.124), and platelets (169.2 vs 166.5; respectively; P = 0.369; Table 1). Also, inflammatory biomarkers, such as ESR (32.4 vs. 33.1; respectively; P = 0.485), CRP (16.3 vs. 14.9; respectively; P = 0.241), and ferritin (365.2 vs. 375.3; respectively; P = 0.399) showed similar increases in the two high- and low-dose groups (Table 1). During hospitalization, some patients in both the high- and low-dose methylprednisolone groups received remdesivir and tocilizumab. However, there were no significant differences between the groups in terms of the number of patients receiving remdesivir (18.7% vs. 17.0%; respectively; P = 0.829) and tocilizumab (27.0% vs. 12.8%; respectively; P = 0.081), as well as the average dosage of these medications (5.06 vs. 4.67 mg; P = 0.103 for the remdesivir dose, and 0.73 vs. 0.53 mg; P = 0.933 for the tocilizumab dose; Table 2). Moreover, the number of patients who required hemoperfusion (16.6% vs. 11.3%; respectively; P = 0.395), as well as the sessions of hemoperfusion, were similar in the high- and low-dose groups (0.91 vs 0.61; respectively; P = 0.756; Table 2). 

**Table 2.** Therapeutics and Interventions Received During Admission Due to COVID-19 Pneumonia ^a^

Therapeutic	High-Dose Methylprednisolone (N = 48)	Low-Dose Methylprednisolone (N = 47)	P Value
**Received remdesivir**	9 (18.7)	8 (17.0)	0.829
**Remdesivir dose (mg)**	5.06 ± 1.17	4.67 ± 0.935	0.103
**Received tocilizumab**	13 (27.0)	6 (12.8)	0.081
**Tocilizumab dose (mg)**	0.73 ± 0.4	0.53 ± 0.19	0.933
**Received hemoperfusion**	8 (16.6)	5 (11.3)	0.395
**Hemoperfusion sessions**	0.91 ± 0.35	0.61 ± 0.19	0.756
**Requirement for invasive ventilation**	14 (29.2)	17 (36.2)	0.466
**Invasive ventilation (days)**	3.77 ± 1.91	2.09 ± 1.05	0.250
**Requirement non-invasive ventilation**	8 (16.6)	11 (23.4)	0.409
**Non-invasive ventilation (days)**	1.47 ± 0.25	1.1 ± 0.47	0.412
**Requirement for ICU admission**	34 (70.8)	27 (57.4)	0.124
**ICU stay (days)**	5.44 ± 4.65	4.52 ± 4.92	0.356
**Hospital stay (days)**	8 ± 3.19	6.91 ± 3.42	0.114
**Oxygen support with mask (days)**	5.90 (3.07)	5.17 (3.49)	0.285
**Oxygen saturation upon discharge**	87.19 ± 3.83	86.64 ± 3.83	0.593

Abbreviation: ICU, intensive care unit.

^a^ Values are expressed as Mean ± SD or No. (%).

No significant difference was found in either the rate of patients who required invasive ventilation (29.2% vs 36.2%, respectively; P = 0.466) or the duration of intubation among those who were under invasive ventilation in the high- and low-dose groups (3.77 vs 2.09 days; respectively; P = 0.25). Similarly, patients receiving high-dose methylprednisolone had close rates of being under non-invasive ventilation compared with the low-dose methylprednisolone group (16.6% vs 23.4%, respectively; P = 0.409). Of note, the number of patients transferred to intensive care units (ICUs; 70.8% vs. 57.4%; respectively; P = 0.124), the length of ICU stays (5.44 vs. 4.52 days; respectively; P = 0.356), and the total duration of hospital stay (8 vs. 6.91 days; respectively; P = 0.114) were all statistically similar in the high- and low dose groups. After being eligibly discharged, patients of the high- and low-dose groups had similar oxygenic saturation (87.19 ± 3.83% vs. 86.64 ± 3.83%, respectively; P = 0.5; Table 2). Bacterial pneumonia and septic shock, as in-hospital complications, were evaluated in patients. Whilst the rate of septic shock did not have a remarkable difference between the high- and low-dose groups (4.2% vs. 6.4%, respectively; P = 0.629), patients who were under 72-hour 1000 mg pulse of methylprednisolone before regular dosage therapy were reported to develop considerably more bacterial pneumonia co-infection (18.7% vs. 10.6%; respectively; P = 0.01). However, one must notice that eventually, the rate of fatality did not differ between the high- and low-dose groups (29.2% vs 38.3%, respectively; P = 0.346; Table 3). 

**Table 3.** Secondary Events During Hospitalization Due to COVID-19 Pneumonia ^a^

Events	High-Dose Methylprednisolone (N = 48)	Low-Dose Methylprednisolone (N = 47)	P Value
**Septic shock**	2 (4.2)	3 (6.4)	0.629
**Bacterial pneumonia**	9 (18.7)	5 (10.6)	0.018
**Mortality**	14 (29.2)	18 (38.3)	0.346

^a^ Values are expressed as No. (%).

## 5. Discussion

The trial began in April of 2022, during a period when Iran was experiencing a significant wave of COVID-19. At that time, the potential role of corticosteroids in treating the disease had not yet been emphasized. Thus, the exact role and dose of corticosteroids were unclear. The results of this research showed that patients with mild to moderate ARDS receiving 1000 mg/day pulse therapy of methylprednisolone before starting 1 mg/kg/12 hours methylprednisolone did not experience any increased survival, decreased ICU stay and overall hospital stay or improved rates of mechanical ventilation or non-invasive ventilation (NIV). However, the risk of bacterial pneumonia co-infection was considerably greater in the group receiving large doses of methylprednisolone.

The 2 effective evidence-based treatments that are now in widespread use for hospitalized patients with COVID-19 are systematic corticosteroids and remdesivir (9, 11, 14). In fact, among immune-modulating agents, steroids have been found to slow the development of respiratory failure and mortality in cases of severe COVID-19 pneumonia with cytokine storm syndrome (7, 9).

Glucocorticoids exert their suppressive effects on the human immune system by preventing macrophages from performing their phagocytic roles and by reducing the activity and quantity of T cells while having little to no effect on humoral immunity (15). Rapid Evidence Appraisal for COVID-19 Therapies (REACT), the WHO’s biggest meta-analysis of clinical studies, has shown that systemic steroid therapy among severe COVID-19 patients is helpful in lowering in-hospital mortality (12, 16).

The WHO has issued recommendations for the use of low-dose steroid therapy in the treatment of severe COVID-19 patients, emphasizing their advantages in reducing mortality and the requirement for mechanical ventilation; while also advising against the use of it in mild to moderate cases (16). Other studies have shown that early steroid treatment could lower mortality, decrease the number of days that ARDS patients require invasive ventilation, and increase the number of days when organ support is not required (9-12, 15, 17, 18). These findings demonstrate once more how systemic steroid therapy might increase the likelihood of survival.

Nevertheless, up until recently, there is no strong consensus agreement on the standards for the amount of steroid administration for COVID-19 patients, and most of the data showing the advantages of corticosteroids in COVID-19 came from observational studies rather than clinical trial studies (19, 20). Consistent with our findings, previous retrospective observational studies have shown that glucocorticoid pulse treatment does not appear to be more advantageous than lowering dosages in COVID-19 (21). Moreover, Jeronimo et al. (22) conducted research on COVID-19 patients with ARDS who received methylprednisolone and found a high mortality rate of around 30%, compared with patients who received low-dose treatment with a fatality rate of 18%. Other studies have found that individuals with comorbid conditions and older age are at higher risk of death while taking high doses of methylprednisolone (23, 24).

On the other hand, some research has supported the use of methylprednisolone in critically ill patients with severe COVID-19 (25). In instances of severe SARS-CoV-2 pneumonia treated with 1 to 2 mg/kg/day of methylprednisolone over the course of 7 days, You et al. (26) principally observed a quicker improvement of oxygen saturation and a shorter duration of fever. Further, high-dose methylprednisolone was found to be superior to dexamethasone in studies by Pinzón et al. (25) and Ranjbar et al. (27) in improving clinical conditions and decreasing the need for invasive ventilation. The fact that methylprednisolone could reach into the lungs more extensively rather than dexamethasone may be the cause of this advantageous impact, which would make it more effective in improving lung compliance. Although, in the aforementioned investigations, the mortality rate improvement was not statistically significant. Similarly, several investigations have shown that early methylprednisolone therapy could improve clinical outcomes in hypoxic individuals with more severe illnesses (28).

It is worth noting that among various types of corticosteroids, we chose to administer methylprednisolone to COVID-19 patients who met the criteria for treatment. Methylprednisolone is a readily accessible, inexpensive corticosteroid that has been used more frequently than other corticosteroids in ARDS studies. Methylprednisolone, an intermediate-acting medication, has a 5-fold potency advantage over short-acting medications such as hydrocortisone. While dexamethasone, a long-acting medication, has a 25-fold advantage over short-acting medications and has been used in several settings due to the COVID-19 pandemic (25, 29). Additionally, because methylprednisolone has little to no mineralocorticoid action, it will not increase the risk of fluid retention (a sodium/water mismatch), which is typically found in severe ARDS cases (30).

Our findings revealed that individuals receiving high doses of steroids were at greater risk of developing concurrent bacterial pneumonia. Observational data currently available point to a greater risk of subsequent fungal or bacterial infections after corticosteroid usage in viral syndromes (as was previously seen in influenza) (31), as well as in compromised immune responses to respiratory syncytial virus (RSV) (8, 32). In our study, individuals who received methylprednisolone did not have a higher incidence of septic shock. All patients were hospitalized and taking a macrolide and ceftriaxone at the same time, which may have complicated the accurate assessment of this possible adverse effect of corticosteroid treatment. It should be noted that in the treatment of septic shock, corticosteroid medications have been used to increase systemic vascular resistance and enhance mineralocorticoid function, with the goal of restoring effective blood volume (33, 34).

Regarding the absolute efficacy of corticosteroids in COVID-19, numerous issues remain unresolved. Notably, a recent systematic analysis of corticosteroid trials revealed that MERS-CoV-1 and SARS-CoV-1 had delayed viral clearance. Due to the potential for increased viral shedding after steroid treatment is discontinued, it is advisable to continue such medications for more than 5 days or until clinical improvement is observed, particularly when initiated early in the course of illness. Although we did not have the opportunity to assess this consequence in our investigation, high-dose corticoids have been demonstrated to affect long-term viral shedding (34). However, this theory needs to be validated, and future studies should include longer virologic follow-ups.

This study had several strengths, including being conducted in a public hospital setting that adhered to appropriate clinical practices. Additionally, it was designed to specifically evaluate the role of adjuvant steroid pulse therapy, which has been rarely assessed in previous literature (13). However, the study also had several limitations, including (1) a relatively small sample size, which limited our ability to more accurately evaluate minor differences between the case and control groups; (2) a single-center setting; (3) a relatively high overall fatality rate (33.7%) compared to other similar studies, which may be partially explained by the participant demographics and the higher prevalence of co-morbidities; and 4) a lack of data to estimate the impact of these treatment regimens on leading complications of COVID-19, such as pulmonary fibrosis.

### 5.1. Conclusions

The use of methylprednisolone pulse therapy during the first 3 days of hospitalization, before initiating 1 mg/kg/12 hours methylprednisolone, was not sufficient to improve the prognosis, hospital events, and final outcomes in COVID-19 patients. Our analysis also showed that pulse therapy with methylprednisolone increased bacterial co-infection pneumonia in hospitalized patients with COVID-19. Further studies are needed to determine whether clinicians should consider increasing the dosage of steroids in the treatment of COVID-19 or if caution is warranted due to the increased risk of concurrent bacterial pneumonia. Further, more evaluations of the prolonged adverse effects, which are frequently dose-dependent, could help clinicians to decide between regimens if both of the dosing formulations are verified to have equivalent efficacies in clinical outcomes.

## Clinical Trial Registration Code:

IRCT20130917014693N13.

## References

[1] Gharebaghi N, Farshid S, Boroofeh B, Nejadrahim R, Mousavi J, Dindarian S (2021). Evaluation of epidemiology, clinical features, prognosis, diagnosis and treatment outcomes of patients with covid-19 in West Azerbaijan Province.. Int J Clin Pract..

[2] Rahimi FS, Afaghi S, Esmaeili Tarki F, Goudarzi K, Malekpour Alamdari N (2020). Viral outbreaks of sars-cov1, sars-cov2, mers-cov, influenza h1n1, and ebola in 21st century; a comparative review of the pathogenesis and clinical characteristics.. Sch Med Stud J..

[3] Al-Dorzi HM, Aldawood AS, Almatrood A, Burrows V, Naidu B, Alchin JD (2021). Managing critical care during covid-19 pandemic: The experience of an ICU of a tertiary care hospital.. J Infect Public Health..

[4] Besharat S, Rahimi F, Afaghi S, Esmaeili Tarki F, Pourmotahari F, Fathi M (2021). Chest CT imaging characteristics of covid-19 pneumonia in surviving and non-surviving hospitalized patients: A retrospective study in a referral center in Tehran, Iran.. Iran J Radiol..

[5] Dastan F, Nadji SA, Saffaei A, Tabarsi P (2020). Tocilizumab administration in a refractory case of covid-19.. Int J Antimicrob Agents..

[6] Alamdari NM, Afaghi S, Rahimi FS, Tarki FE, Tavana S, Zali A (2020). Mortality risk factors among hospitalized covid-19 patients in a major referral center in iran.. Tohoku J Exp Med..

[7] Afaghi S, Esmaeili Tarki F, Rahimi FS, Naghibi Irvani SS, Besharat S, Malekpour Alamdari N (2020). Therapeutic options and critical care strategies in covid-19 patients; where do we stand in this battle?. Sch Med Stud J..

[8] Lee CCE, Ali K, Connell D, Mordi IR, George J, Lang EM (2021). Covid-19-associated cardiovascular complications.. Diseases..

[9] Johns M, George S, Taburyanskaya M, Poon YK (2022). A review of the evidence for corticosteroids in covid-19.. J Pharm Pract..

[10] Mishra GP, Mulani J (2021). Corticosteroids for covid-19: The search for an optimum duration of therapy.. Lancet Respir Med..

[11] Wagner C, Griesel M, Mikolajewska A, Mueller A, Nothacker M, Kley K (2021). Systemic corticosteroids for the treatment of covid-19.. Cochrane Database Syst Rev..

[12] Keyt H (2021). WHO recommends corticosteroids for patients with severe or critical covid-19.. Ann Intern Med..

[13] Edalatifard M, Akhtari M, Salehi M, Naderi Z, Jamshidi A, Mostafaei S (2020). Intravenous methylprednisolone pulse as a treatment for hospitalised severe covid-19 patients: Results from a randomised controlled clinical trial.. Eur Respir J..

[14] Jain S, Bala M, Sachdeva HC, Talwar V, Ganapathy U (2021). A retrospective evaluation of combination therapy of methylprednisolone and remdesivir for severe covid-19 patients.. J Clin Diagnostic Res..

[15] Williams DM (2018). Clinical pharmacology of corticosteroids.. Respir Care..

[16] Sterne JA, Murthy S, Diaz JV, Slutsky AS, Villar J, Angus DC (2020). Association between administration of systemic corticosteroids and mortality among critically ill patients with covid-19: A meta-analysis .. Jama..

[17] De Backer D, Azoulay E, Vincent JL (2020). Corticosteroids in severe covid-19: A critical view of the evidence.. Crit Care..

[18] Sarma P, Bhattacharyya A, Kaur H, Prajapat M, Prakash A, Kumar S (2020). Efficacy and safety of steroid therapy in covid-19: A rapid systematic review and meta-analysis.. Indian J Pharmacol..

[19] Ambrosino N, Vitacca M (2018). The patient needing prolonged mechanical ventilation: a narrative review.. Multidiscip Respir Med..

[20] Nasa P, Chaudhry D, Govil D, Daga MK, Jain R, Chhallani AA (2021). Expert consensus statements on the use of corticosteroids in non-severe covid-19.. Indian J Crit Care Med..

[21] Fernandez-Cruz A, Ruiz-Antoran B, Munoz-Gomez A, Sancho-Lopez A, Mills-Sanchez P, Centeno-Soto GA (2020). A retrospective controlled cohort study of the impact of glucocorticoid treatment in sars-cov-2 infection mortality.. Antimicrob Agents Chemother..

[22] Jeronimo CMP, Farias MEL, Val FFA, Sampaio VS, Alexandre MAA, Melo GC (2021). Methylprednisolone as adjunctive therapy for patients hospitalized with coronavirus disease 2019 (covid-19; metcovid): A randomized, double-blind, phase iib, placebo-controlled trial.. Clin Infect Dis..

[23] Monreal E, Sainz de la Maza S, Natera-Villalba E, Beltran-Corbellini A, Rodriguez-Jorge F, Fernandez-Velasco JI (2021). High versus standard doses of corticosteroids in severe covid-19: A retrospective cohort study.. Eur J Clin Microbiol Infect Dis..

[24] Chen Y, Li L (2021). Influence of corticosteroid dose on viral shedding duration in patients with covid-19.. Clin Infect Dis..

[25] Pinzon MA, Ortiz S, Holguin H, Betancur JF, Cardona Arango D, Laniado H (2021). Dexamethasone vs methylprednisolone high dose for covid-19 pneumonia.. PLoS One..

[26] You X, Wu CH, Fu YN, He Z, Huang PF, Chen GP (2020). The use of methylprednisolone in covid-19 patients: A propensity score matched retrospective cohort study.. PLoS One..

[27] Ranjbar K, Moghadami M, Mirahmadizadeh A, Fallahi MJ, Khaloo V, Shahriarirad R (2021). Methylprednisolone or dexamethasone, which one is superior corticosteroid in the treatment of hospitalized COVID-19 patients: a triple-blinded randomized controlled trial.. BMC Infect Dis..

[28] Fadel R, Morrison AR, Vahia A, Smith ZR, Chaudhry Z, Bhargava P (2020). Early short-course corticosteroids in hospitalized patients with covid-19.. Clin Infect Dis..

[29] Corral-Gudino L, Bahamonde A, Arnaiz-Revillas F, Gomez-Barquero J, Abadia-Otero J, Garcia-Ibarbia C (2021). Methylprednisolone in adults hospitalized with covid-19 pneumonia : An open-label randomized trial (glucocovid).. Wien Klin Wochenschr..

[30] Ocejo A, Correa R (2023). Methylprednisolone.. StatPearls..

[31] Ni YN, Chen G, Sun J, Liang BM, Liang ZA (2019). The effect of corticosteroids on mortality of patients with influenza pneumonia: a systematic review and meta-analysis.. Crit Care..

[32] Rafiullah M, Siddiqui K (2020). Corticosteroid use in viral pneumonia: experience so far and the dexamethasone breakthrough in coronavirus disease-2019.. J Comp Eff Res..

[33] Patel GP, Balk RA (2012). Systemic steroids in severe sepsis and septic shock.. Am J Respir Crit Care Med..

[34] Sadaka F, Grady J, Organti N, Donepudi B, Korobey M, Tannehill D (2020). Ascorbic acid, thiamine, and steroids in septic shock: Propensity matched analysis.. J Intensive Care Med..

